# A comparison of variation between a MHC pseudogene and microsatellite loci of the little greenbul (*Andropadus virens*)

**DOI:** 10.1186/1471-2148-5-47

**Published:** 2005-09-13

**Authors:** Andres Aguilar, Thomas B Smith, Robert K Wayne

**Affiliations:** 1 Department of Ecology and Evolutionary Biology, University of California, Los Angeles, CA 90095 USA; 2 Center for Tropical Research, Institute of the Environment, 1609 Hershey Hall, University of California, Los Angeles, CA 90095 USA; 3 Southwest Fisheries Science Center & Department of Ocean Sciences, 110 Shaffer Road, University of California, Santa Cruz, California 95060 USA

## Abstract

**Background:**

We investigated genetic variation of a major histcompatibility complex (MHC) pseudogene (*Anvi*-DAB1) in the little greenbul (*Andropadus virens*) from four localities in Cameroon and one in Ivory Coast, West Africa. Previous microsatellite and mitochondrial DNA analyses had revealed little or no genetic differentiation among Cameroon localities but significant differentiation between localities in Cameroon and Ivory Coast.

**Results:**

Levels of genetic variation, heterozygosity, and allelic diversity were high for the MHC pseudogene in Cameroon. Nucleotide diversity of the MHC pseudogene in Cameroon and Ivory Coast was comparable to levels observed in other avian species that have been studied for variation in nuclear genes. An excess of rare variants for the MHC pseudogene was found in the Cameroon population, but this excess was not statistically significant. Pairwise measures of population differentiation revealed high divergence between Cameroon and Ivory Coast for microsatellites and the MHC locus, although for the latter distance measures were much higher than the comparable microsatellite distances.

**Conclusion:**

We provide the first ever comparison of variation in a putative MHC pseudogene to variation in neutral loci in a passerine bird. Our results are consistence with the action of neutral processes on the pseudogene and suggest they can provide an independent perspective on demographic history and population substructure.

## Background

Portrayed as the paradigm of neutral evolution [1], pseudogenes are thought be free of selective forces that constrain functional genes and this single feature should make pseudogenes highly attractive for population genetic studies. Pseudogenes may be more appealing than introns for population genetic studies, as introns may be closely linked to functional gene regions [2] and therefore may often be under the influence of selection [3]. Though pseudogenes have been the focus of molecular evolutionary studies at the species level, there is a paucity of research that utilizes them for analysis of populations [see [4,5]]. The main reason for the lack of pseudogenes in population level studies may be that few have been isolated for non-model taxa.

Levels of population differentiation and variability depend on the type of molecular marker used. Modes of inheritance [6], mutation rate [7], mutation models [8,9], recombination [10], and natural selection [11] are important factors that can affect estimates of genetic variability, and consequently measures of population differentiation [12]. Microsatellites are 2–5 base pair (bp) repetitive elements found throughout eukaryotic genomes and are hypervariable genetic markers that are commonly used in molecular genetic studies of natural populations [7]. The use of nuclear sequences in population genetic studies is becoming more common in evolutionary studies [13-15]. However, nuclear sequences are often not as attractive for population genetic studies as they generally have much lower mutation rates than microsatellite loci and consequently are less variable. Most recent population genetic studies have utilized non-coding nuclear markers such as microsatellites or nuclear length variants such as amplified fragment length polymorphisms [15-17,19].

The little greenbul (*Andropadus virens*) is a small passerine that inhabits rainforests in Sub-Saharan Africa [20]. Previous research on the little greenbul with di- and tetra-nucleotide microsatellite loci has revealed extensive gene flow among Cameroon localities [21,22] and showed Cameroon and Ivory Coast populations to be genetically distinct [22]. Analysis of mitochondrial DNA control region variation found Cameroon and Ivory Coast populations define two distinct sequence clades [23]. These phylogeographic units correspond to putative rainforest refugia in lower (Cameroon) and upper (Ivory Coast) Guinea [23,24].

We assessed genetic variation in *Anvi*-DAB1, a putative MHC pseudogene in the little greenbul. This designation was based on the presence of frame-shift mutations within the reading frame of exon 2 (Aguilar et al., in review). Genetic variation in *Anvi*-DAB1 should be correlated with that of neutral loci. To test this prediction, we compared genetic variation in *Anvi*-DAB1 to variation in six microsatellite loci in little greenbuls from Cameroon and Ivory Coast.

## Results

We sequenced 16 individuals from Ivory Coast and 55 individuals from Cameroon for variation in the *Anvi*-DAB1 MHC gene. A total of 17 alleles were found (Table 1) and three alleles, Anvi-DAB1*07, Anvi-DAB1*08, and Anvi-DAB1*14, were shared between Cameroon and Ivory Coast. Eleven of 17 alleles were unique to Cameroon and three were unique to Ivory Coast. Cameroon exhibited much more allelic diversity than Ivory Coast for *Anvi*-DAB1 (Table 1). An allele previously found containing a frame shift mutation (Anvi-DAB1*05) was found at a frequency of 0.23 in Nkwouak and 0.03 in Ndibi. Observed heterozygosity (H_o_) for the Cameroon sites varied from 0.30 (Wakwa) to 1.0 (Tibati) for *Anvi*-DAB1 (Table 1). Two of the Cameroon populations, Ndibi and Wakwa, exhibited significant deviations from H-W equilibrium (p < 0.05) for the *Anvi*-DAB1 locus. Per site nucleotide diversity (π) for Cameroon and Ivory Coast was 0.007 and 0.004 (Table 1). The number of segregating sites (S) for the 14 and 4 alleles found in Cameroon and Ivory Coast was 13 and 3, respectively (Table 1). Within Cameroon, the Ndibi site possessed the greatest number of alleles (k = 11) and nucleotide diversity was 0.006 or 0.007 at each site (Table 1).

**Table 1:** Observed (H_o_) and expected (H_e_) heterozygosities, allelic richness (A) for the *Anvi*-DAB1 and 6 microsatellite loci. Mean allelic richness (A) is give for the 6 microsatellite loci. DNA sequence polymorphism statistics are reported for the *Anvi*-DAB1 gene (nucleotide diversity – Π; Tajima;s D – D; Fu and Li's F* – F).

	Microsatellites						*Anvi*-DAB1				
	H_o_	H_e_	A	H_o_	H_e_	A	k	S	Π	D	F*
Cameroon							11	13	0.007	-0.84	-1.42
Luna	0.51	0.62	6.8	0.38	0.57	5.0	5	5	0.006	-0.03	0.37
Nkwouak	0.70	0.68	6.8	0.65	0.77	5.7	8	9	0.007	-0.36	-1.05
Ndibi	0.62	0.62	6.4	0.71	0.79	7.1	11	9	0.007	-0.82	-1.86
Wakwa	0.60	0.66	7.1	0.30	0.69	5.7	6	6	0.006	-0.36	-0.83
Ivory Coast	0.42	0.55	5.5	0.41	0.71	4.7	6	9	0.007	-0.76	-0.05

Tajima's D and Fu and Li's F* were both negative for the pooled Cameroon (D = -0.885 and F = -1.330; Table 1) and Ivory Coast sample (D = -0.431 and F* = -0.798; Table 1). However, these values were not significantly different from zero (p > 0.1). All of the sites sampled possessed negative values of Tajima's D (Table 1) whereas all but one of the sites had a negative value for Fu and Li's F* (Luna; Table 1). None of the values for Tajima's D or Fu and Li's F* were significantly different from zero.

Pairwise measures of population divergence based on *Anvi*-DAB1 and microsatellite data were in general agreement (Table 2). For microsatellite loci and *Anvi*-DAB1, Ivory Coast had the highest degree of differentiation from all other populations (Table 2). All pairwise F_ST _measures for Anvi-DAB1allelic data between Ivory Coast and Cameroon site were statistically greater than zero (Table 2). Likewise, all four pairwise F_ST _measures between Ivory Coast and Cameroon sites for the six microsatellite loci were significantly greater than zero (Table 2). However, F_ST _values between Cameroon and the Ivory Coast were larger for *Anvi*-DAB1 (allelic: 0.222 +/- 0.06 [s.d.]; sequence: 0.236 +/- 0.04 [s.d.]) than for the six microsatellite loci (0.086 +/- 0.01 [s.d.]). In contrast, the mean F_ST _for all pairwise comparisons within Cameroon is lower for *Anvi*-DAB1 allelic (0.004 +/- 0.03 [s.d.]) than for microsatellite data (allelic: 0.012 +/- 0.01 [s.d.]) whereas sequence data has the highest levels of F_ST _(0.026 +/- 0.01 [s.d.]). The F_ST _measures from microsatellites were not significantly different from *Anvi*-DAB1 allelic (p = 0.23; t = -1.24) and *Anvi*-DAB1 sequence F_ST _(p = 0.08; t = -1.82). However, all pairwise values within Cameroon are low suggesting high rates of gene flow. The results of the linkage disequilibrium (LD) test for recent divergence versus ongoing gene flow are all positive, indicating that gene flow is most likely occurring among the sampled sites within Cameroon (Table 3). Allelic richness was not highly correlated between the two marker types (r^2 ^= 0.11).

**Table 2:** Measures of pairwise population differentiation for the 6 microsatellite loci (above diagonal) and for *Anvi*-DAB1 (below diagonal). For the *Anvi*-DAB1 F_ST _measures, the numbers on top are based on allelic data and the numbers on bottom are based on sequence data (see Methods). Numbers in bold indicate measures that are significantly different from zero (see Methods).

	Luna	Nkwouak	Ndibi	Wakwa	Ivory Coast
Luna	-	**0.016**	**0.021**	0.0	**0.080**
Nkwouak	0.036 0.039	-	**0.027**	0.008	**0.101**
Ndibi	0.036 0.037	0.019 0.034	-	0.003	**0.084**
Wakwa	-0.041 0.027	-0.003 0.001	-0.024 0.016	-	**0.077**
Ivory Coast	**0.301** 0.285	**0.229** 0.214	**0.168** 0.196	**0.191** 0.248	-

**Table 3:** Results of the linkage disequilibrium test for ongoing gene flow versus recent divergence among Cameroon sites for the *Anvi*-DAB1 locus. Reference group for comparison is listed in rows. NA indicated that less than four pairs of sites were compared and means were not taken.

Reference Population	Luna	Ndibi	Nkwouak	Wakwa
Luna	-	1.167	1.167	1.167
Ndibi	1.167	-	0.762	0.733
Nkwouak	0.867	0.381	-	0.400
Wakwa	1.167	NA	NA	-

Pairwise values of F_ST _for allelic and sequence Anvi-DAB1 information were highly correlated (r^2 ^= 0.944) and both statistics were correlated with values of F_ST _for microsatellite loci (r^2 ^= 0.889, r^2 ^= 0.876, respectively). However, none of these relationships were significantly based on the Mantel's test likely reflecting the small number of matrix entries (n = 4). The Mantel's test was also preformed omitting the pairwise measures from Lamto, and a non-significant positive correlation was still found (r^2 ^= 0.864; p = 0.167).

**Figure 1:**
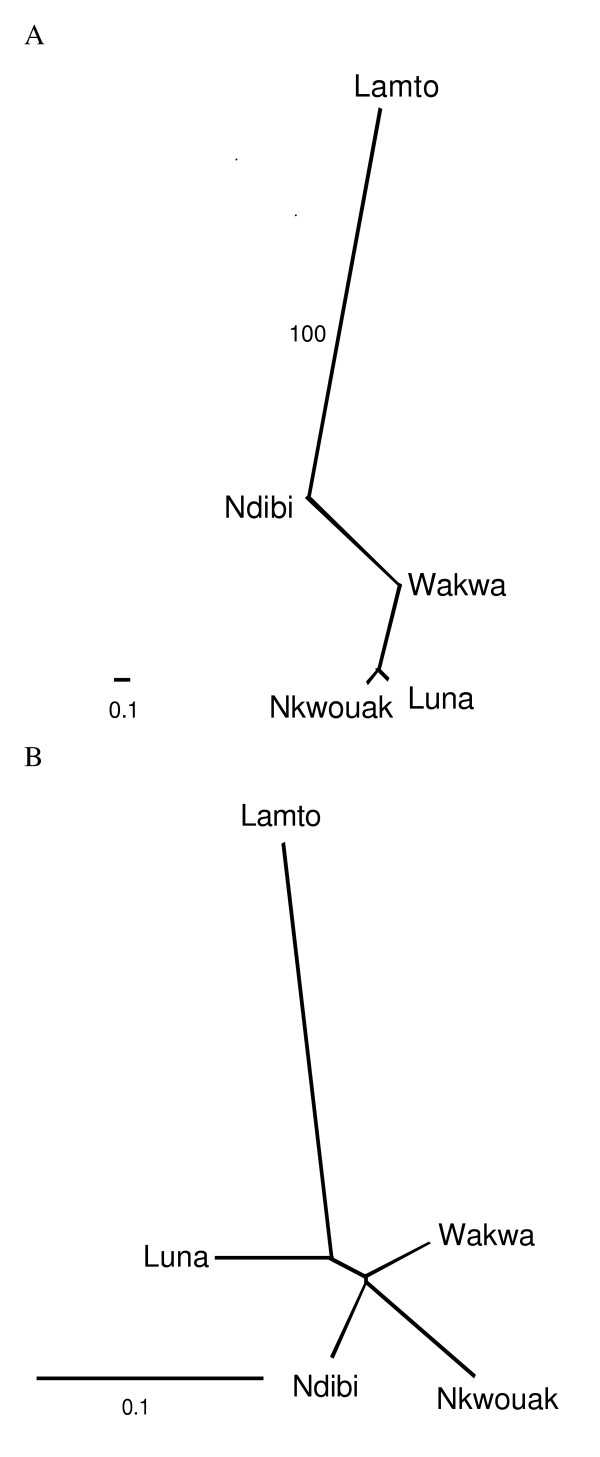
Unrooted neighbor-joining trees for the five *A. virens *populations using Nei's standard distance (Ds) for the allelic data from the *Anvi*-DAB1 locus (A) and 6 microsatellite loci (B). Bootstrap support above 50% is shown (see methods).

Population level relationships based on genetic distance measures varied with distance measure used and with locus type (Figure 1). All neighbor-joining trees showed that Ivory Coast is divergent from the Cameroon sites (Figure 1). However, high bootstrap support distinguishing Ivory Coast from Cameroon is only evident in the tree constructed using D_S _with *Anvi*-DAB1 sequence data (Figure 1B). There was not any support within trees or consistency among trees with regard to the relationships among the Cameroon sites (Figure 1).

## Discussion

We have shown that measures of population differentiation for a MHC pseudogene, *Anvi*-DAB1, are not significantly differently different from those of six unlinked neutral microsatellite loci. A high degree of differentiation for the *Anvi*-DAB1 pseudogene as measured by F_ST _was found between sites in Cameroon and Ivory Coast, a result that has been previously found in studies that utilized microsatellite loci [22], and mitochondrial DNA [23]. Within sampled Cameroon sites, high levels of gene flow, as evidenced by low pairwise F_ST _measures, was found for the *Anvi*-DAB1 locus. This again is concordant with results from microsatellite and mitochondrial DNA [21-23].

Evidence for *Anvi*-DAB1 being a pseudogene is based on the observation that an allele containing a frame-shift mutation (*Anvi*-DAB1*05) is homozygous in three individuals, nearly equal rates of synomonous and nonsynomonous substitutions, absence from a survey of transcribed genes in the little greenbul, high divergence in sequence type when compared to classical transcribed MHC sequences, and a lack of conserved MHC class II vertebrate amino acid residues (Aguilar et al., in review). Pseudogenes are rarely used in studies of natural populations, yet they may be valuable tool for quantifying genetic variation and differentiation. For example, polymorphism at the psGBA pseudogene in humans was found to be concordant with previous studies of neutral genes [5]. Nucleotide diversity in Anvi-DAB1 was found to be low, and was similar to that found for another avian MHC pseudogene (*Came*-DAB1: π = 0.03 [38]). This level of polymorphisms is also low compared to functional MHC genes isolated from other birds and vertebrate taxa [25]. However, study of a human MHC class I pseudogene (HLA-H) found elevated levels of genetic variation, and this was attributed to the linkage of HLA-H to functional HLA loci [26]. Therefore, although pseudogenes maybe useful loci in population genetic studies, comparison of their genetic variability to neutral markers is needed to determine if levels of genetic variability may be influenced by selection.

Negative D and F* values suggest an excess of rare mutants in the pooled Cameroon population though these values were not statistically significant. Similarly, all individual sites possessed negative values for Tajima's D and Fu and Li's F* but again these were not statistically significant. An overabundance of rare mutants in a sample can be caused by recent population expansions [27,28], selective sweeps [29,30], or from pooling samples [31,32]. Further sampling of sites within Cameroon and Ivory Coast, the inclusion of other loci, and establishing fine-scale patterns of population structure will elucidate the significance of the excess of rare mutants for the *Anvi*-DAB1 gene.

Levels of differentiation were high and significant between Ivory Coast and Cameron for the MHC pseudogene (allelic and sequence data) and microsatellite loci suggesting long-term isolation. In contrast, low mean F_ST _for within Cameroon comparisons, for both *Anvi*-DAB1 and microsatellites, is indicative of a high level of gene flow among the sampled Cameroon sites. The results of the LD test, coupled with the low F_ST _values, indicate that gene flow is still occurring within the samples Cameroon sites. The positive correlation of pairwise measures of population differentiation for *Anvi*-DAB1 and the six microsatellite loci and the lack of significant differences in levels of within and between population variation supports drift and migration are the primary influences on the observed genetic variation at *Anvi*-DAB1.

The lack of any significant correlation between allelic richness measures from microsatellites and the MHC gene could be due to the small-observed differences in allelic richness across populations and/or the low number of populations sampled. High gene flow, as well as large effective population sizes, could account for low discrepancy in allelic richness. To determine if drift is an important factor affecting allelic richness at the two marker sets we would need to sample populations with low effective population size, where we would expect a concordant decrease in microsatellites and MHC variation.

Null alleles could account for the deficiency of heterozygotes observed in many samples. Other factors that could contribute to the deviations from Hardy-Weinberg expectations include sampling artifacts, family structure, and non-random mating. Further work that could elucidate the role of null alleles in generating the observed pattern in heterozygosity would include the re-designing of PCR primers and the use of less stringent PCR conditions. However, such modifications could lead to the amplification of non-orthologous closely related loci.

The unrooted neighbor-joining dendrograms showed that Ivory Coast was topologically distinct from Cameroon localities for both *Anvi*-DAB1 and microsatellite data. The main difference in the neighbor-joining trees was the degree of genetic distance observed for both marker types, as the *Anvi*-DAB1 dendrogram constructed with Ds showed much lower differentiation between Ivory Coast and Cameron than the dendrogram based on microsatellite loci (Figure 1). This difference is most likely due to both the limited sample from Ivory Coast and the effect of using a single locus. Unrepresentative allele frequencies as well as the biases associated with a single locus might suggest the average distance measures based on the six microsatellite loci more accurately reflect population history.

The observed genetic differences between Cameroon and Ivory Coast little greenbul populations are a result of geographic isolation two million years ago [23]. Reciprocally monophyletic clades representing the Upper and Lower Guinea refugia were found using mitochondrial NADH dehydrogenase subunit 2 sequence data [23]. The corrected sequence divergence between the two clades was 4.7%, and the estimated time of gene divergence was 2 mya. A more rigorous analysis of 10 microsatellite loci revealed elevated F_ST _between Cameroon and Ivory Coast and these two population groups were also recovered using a Bayesian clustering approach [22]. Examination of population differentiation within Cameroon sites has revealed low levels of gene flow among lowland forest sites [21-23]. Similar results, based on *Anvi*-DAB1, indicate that this locus is reflecting historical population separations and the contemporary effects of gene flow.

## Conclusion

Comparable measures of population differentiation and similarity in population level phylogenetic trees indicate that the processes that are operating on *Anvi*-DAB1 are analogous to those acting on the typed microsatellite loci. These results suggest that pseudogenes may be useful as molecular tools in population level studies. However, several pseudogenes should be used to decrease locus specific effects and comparisons should be made to other nuclear loci that are unlikely to be under selection (such as microsatellites) so that the influences of selection on pseudogenes can be evaluated. Though pseudogenes may not be as readily available for use, they may become more common as researchers continue large scale sequencing projects on non-model organisms (see [38] and others).

## Methods

Little greebul blood samples were collected by T. B. Smith in Cameroon and Ivory Coast. A total of 71 individuals were genotyped at *Anvi*-DAB1, 55 were from Cameroon, and 16 from Ivory Coast. From Cameroon, localities Luna (n = 8), Nkwouak (n = 20), Ndibi (n = 17), and Wakwa (n = 10) were sampled. The lone site from Ivory Coast was Lamto (n = 16) (see [22] for locality detail). DNA was extracted from blood samples by digestion with proteinase-K followed by phenol-choloroform extraction [33] or by use of a commercially available DNA extraction kit (Qiagen Inc.). The microsatellite dataset used here was from Smith et al. [22] and contained scores on six tetranucleotide microsatellite loci.

The nuclear pseudogene used was the *Anvi*-DAB1 MHC gene isolated from the little greenbul (Aguilar et al. in review). SSCP [34] was used to identify unique alleles. Briefly, both primers were end-labeled with α-^32^P [33] and these radio-labeled primers were used in a PCR reaction with the following conditions: 10 ng genomic DNA, 1 mM of each primer (Anviex2F.1 [TGC CAT GGA CGC TTA CAC T] and Int2R.1 [CCG AGG GGA CAC GCT CT] [35], 1 mM dNTPs, 1 x PCR buffer (Sigma), 0.5 units of Taq polymerase (Sigma) and 1.0 mM MgCl_2 _in a 25 μL reaction volume. These primer pairs target a 267 bp portion of exon 2 from the *Anvi*-DAB1 pseudogene. Reactions were run with the following temperature cycles: an initial 3 min denaturing step at 94°C, 30 sec at 94°C, 30 sec at 58°C, 30 sec at 72°C, and a final 5 min extension at 72°C. Five μL of the PCR reaction were mixed with two μL of stop solution (95% formamide and 0.05% bromophenol blue), heated for 5 min at 95°C then cooled immediately on ice. Two μL of this cocktail were loaded into a 5% non-denaturing polyacrylamide gel containing 5% glycerol (v/v) and run at 20 W for 8–10 hours at room temperature. Gels were transferred to 3 M Whatman paper, dried, and exposed to autoradiographic film for 12–48 hours (depending upon activity of ^32^P). Unique alleles, as identified from SSCP, were isolated from dried gels and re-amplified [34]. PCR products were separated on 1% agarose gels, isolated, and sequenced using forward and reverse primers. Alleles having the same confirmation were sequenced from multiple individuals to assure identity in sequence. Sequencing was done either on an ABI 377 or a Beckman CEQ2000 following manufacture's protocols. Sequences were then imported into SEQUENCHER (GeneCodes, Inc.) and aligned. Sequences have been deposited in [Genbank: AY437894-AY437899; DQ113429-DQ113439].

Observed and expected heterozygosity for *Anvi*-DAB1 and the microsatellite loci were calculated using GENETIX [36]. Deviations from Hardy-Weinberg equilibrium were assessed with the exact test implemented in GENEPOP [37]. We also calculated Tajima's D [38] and Fu and Li's F* [39] for each site and pooled samples from Cameroon to assess any deviations from neutral evolution using DNAsp [40]. Both Tajima's D and Fu and Li's F statistics test for deviations from neutrality by examining the frequency spectra of mutations in the sample. Statistical significance from neutrality was assessed for Tajima's D and Fu and Li's F* using 10,000 coalescent simulations in DNAsp [40].

Pairwise population differentiation (F_ST _or θ) was calculated from allelic data [41] with GENETIX [41] for *Anvi*-DAB1 and for the six microsatellite loci. Significance of pairwise F_ST _measures was assessed with 500 bootstrap replicates in GENETIX. We calculated F_ST _from sequence data using the method of Hudson et al. [42] implemented in DNAsp [40]. The statistical significance of correlations among pairwise measures of F_ST _(for *Anvi*-DAB1 and microsatellite loci) was assessed with a Mantel's test (5000 permutations) using GENETIX. Allelic richness, a measure of allelic variation that takes into account differences in sample sizes among populations, was estimated with the rarefaction method [43]. The rarefaction estimate was based on sampling 16 genes per population.

We used the approach of Machado et al. [44] to distinguish between ongoing gene flow and recent divergence among the Cameroon populations. This method compares the difference in LD between all shared polymorphisms (DSS) between two populations and the LD from pairs of nucleotide sites that are shared between populations and exclusive to one reference population (DSX). This difference has previously been reported as *x*. LD, was estimated as D', and *x *were estimated with the program SITES [45]. Ongoing gene flow is expected to produce positive *x *values, while the lack of gene flow will produce *x *values close to zero [44].

Unrooted neighbor-joining dendrograms also were constructed from genotype data using Nei's standard genetic distance (Ds) [46] calculated between each population pair with the program POPULATIONS [47]. Five hundred bootstrap replicates were preformed to assess the support for branching nodes.

## Authors' contributions

This work started out of a collaborative effort between the laboratories of TBS and RKW. AA designed the study, carried out the laboratory work and statistical analyses, and drafted the manuscript. TBS collected samples and TBS and RKW participated in the design and drafting of the manuscript. All authors read and approved the final manuscript.
